# Addressing uncertainty in genome-scale metabolic model reconstruction and analysis

**DOI:** 10.1186/s13059-021-02289-z

**Published:** 2021-02-18

**Authors:** David B. Bernstein, Snorre Sulheim, Eivind Almaas, Daniel Segrè

**Affiliations:** 1 Department of Biomedical Engineering and Biological Design Center, Boston University, Boston, MA USA; 2 Bioinformatics Program, Boston University, Boston, MA USA; 3 Department of Biotechnology and Food Science, NTNU - Norwegian University of Science and Technology, Trondheim, Norway; 4 Department of Biotechnology and Nanomedicine, SINTEF Industry, Trondheim, Norway; 5 K.G. Jebsen Center for Genetic Epidemiology, NTNU - Norwegian University of Science and Technology, Trondheim, Norway; 6 Department of Biology and Department of Physics, Boston University, Boston, MA USA

## Abstract

The reconstruction and analysis of genome-scale metabolic models constitutes a powerful systems biology approach, with applications ranging from basic understanding of genotype-phenotype mapping to solving biomedical and environmental problems. However, the biological insight obtained from these models is limited by multiple heterogeneous sources of uncertainty, which are often difficult to quantify. Here we review the major sources of uncertainty and survey existing approaches developed for representing and addressing them. A unified formal characterization of these uncertainties through probabilistic approaches and ensemble modeling will facilitate convergence towards consistent reconstruction pipelines, improved data integration algorithms, and more accurate assessment of predictive capacity.

**Supplementary Information:**

The online version contains supplementary material available at 10.1186/s13059-021-02289-z.

## Introduction

Genome-scale metabolic models (GEMs) aim to capture a systems-level representation of the entirety of metabolic functions of a cell. They represent complex cellular metabolic networks using a stoichiometric matrix, which enables sophisticated mathematical analysis of metabolism at the whole-cell level [1]. Not only do GEMs provide a framework for mapping species-specific knowledge and complex ‘omics data to metabolic networks, but coupled with constraint-based reconstruction and analysis (COBRA) methods, such as Flux Balance Analysis (FBA), they facilitate the translation of hypotheses into algorithms that can be used to generate testable predictions of metabolic phenotypes [2–4]. These methods are now used to study biological systems for many different applications, including in metabolic engineering, human metabolism and biomedicine, and microbial ecology [5–11].

Over 100 well-curated GEMs exist for a range of prokaryotes and eukaryotes, offering an organized and mathematically tractable representation of these organisms’ metabolic networks [12, 13]. A detailed protocol has been described for the reconstruction of well-curated GEMs for new organisms [14]. Additionally, the increased availability of whole-genome sequencing in combination with the development of pipelines for automatic model reconstruction has led to several frameworks that support rapid model reconstruction for a large number of non-model organisms [15–19]. For example, the US Department of Energy systems biology knowledgebase (KBase.us) currently enables the automatic generation of draft GEMs from over 80,000 sequenced genomes [20]. Thus, GEMs are rapidly becoming applicable for a wide range of biological applications.

**Fig. 1:**
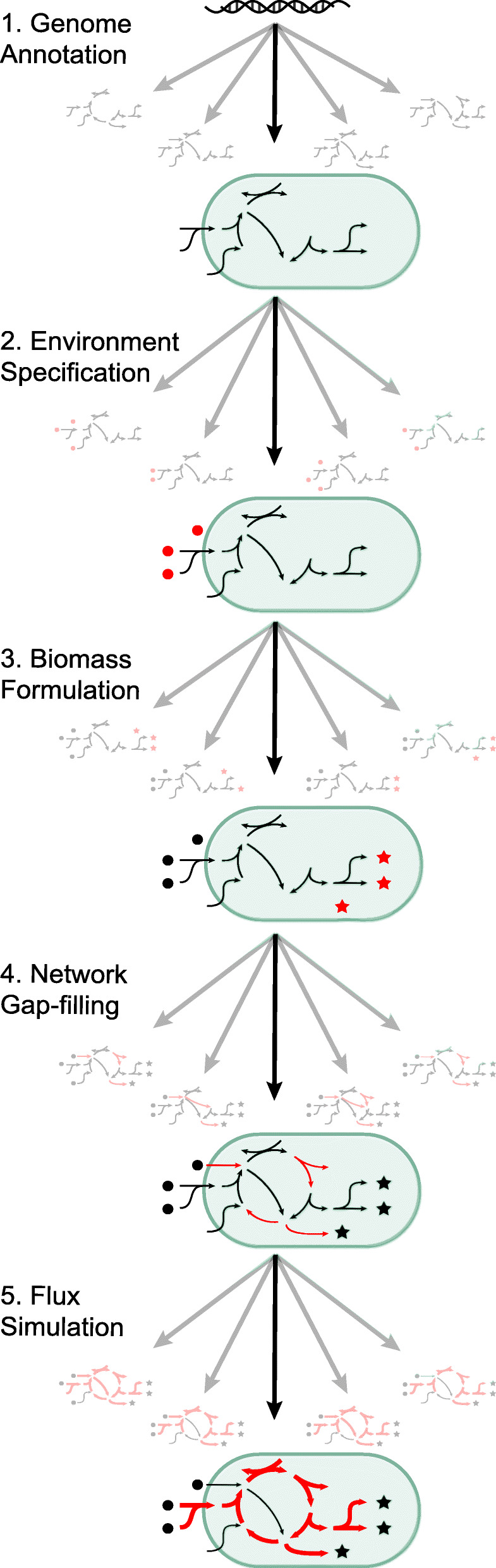
A general progression for genome-scale metabolic model reconstruction and analysis is represented by five major steps. The central black arrows demonstrate a standard approach, which yields a single output from each step. The gray arrows represent the uncertainty in this process, with the output of each step as an ensemble of possible results. The new additions to the model at each step are shown in red: circles represent metabolites, stars represent biomass components, arrows represent metabolic reactions, and bold arrows represent a specific flux distribution

Despite the numerous reconstructions and wide range of applications, GEMs have important limitations [21]. In this review, we focus on one major factor that currently limits the successful application of GEMs: the inherent uncertainty in GEM predictions that arises from degeneracy in both model structure (reconstruction) and simulation results (analysis). While GEM reconstructions typically only yield one specific metabolic network as the final outcome, this one network is indeed one of many possible networks that could have been constructed through different choices of algorithms and availability of information (Fig. 1). The process of GEM reconstruction is divided into (1) genome annotation, (2) environment specification, (3) biomass formulation, and (4) network gap-filling. Different choices in these first four steps can lead to reconstructed networks with different structures (reactions and constraints). On top of these choices, the final phenotypic prediction and biological interpretation is significantly affected by (5) the choice of flux simulation method. This review moves through these five different aspects of GEM reconstruction and analysis, outlining the key sources of uncertainty in each. In addition, we review various approaches that have been developed to deal with this uncertainty. We emphasize approaches that utilize probabilities or an ensemble of models to represent uncertainty. A table associated with each section outlines the different approaches that have been summarized and the sources of uncertainty that they address (Tables 1, 2, 3, 4 and 5).

Our ability to assess and communicate the sources of uncertainty associated with a model can have great impact on the relevance of predictions and on the degree to which these predictions can be constructively used for follow-up studies, as has been noted for the field of systems biology in general [22]. This review is not an introduction to genome-scale metabolic modeling or a survey of its applications, as these topics have been covered elsewhere [5, 11, 23]. Rather, we hope that this text will serve as a roadmap facilitating the development of methods that further formalize a unified characterization of uncertainty in GEM reconstruction and analysis.

## Genome annotation

The first step towards a GEM reconstruction is the identification and functional annotation of the genes encoding metabolic enzymes present in the genome (Table 1). These annotations come from databases that employ homology-based methods for mapping genome sequences to metabolic reactions. The use of these annotation databases in GEM reconstruction pipelines in general is covered in several reviews [24–27]. It has been noted that the choice of a particular database significantly affects the structure of the reconstructed network [19]. This variability can be attributed to the limited accuracy of homology-based methods [28], misannotations present in large databases [29], the fact that many genes can only be annotated as hypothetical sequences of unknown function [30, 31], and the high fraction of “orphan” enzyme functions that cannot be mapped to a particular genome sequence [32]. Some, but not all, of this variability can be mitigated by combining multiple databases to increase the coverage of annotation when reconstructing a GEM [33, 34]. Furthermore, annotation for GEM reconstruction has an added layer of complexity beyond mapping genes to general ontologies or homologs. It is necessary to map genes to the metabolic reactions that they enable. These mappings, referred to as gene-protein-reaction association rules, use Boolean expressions to encode the nonlinear mapping between genes and reactions (manifested in multimeric enzymes, multifunctional enzymes and isoenzymes). The reconstruction and interpretation of these rules adds additional uncertainty to the annotation process. Even if a rule faithfully represents the functional possibilities encoded in a set of genes, the cellular “interpretation” of the rule may be highly nuanced and complex. For example, isoenzymes may not always compensate for each other’s deletion due to different regulatory couplings [35], and alternative usage of the Boolean relationship may best capture the cost of a gene deletion and its degree of evolutionary conservation [36]. An innovative approach for representing gene-protein-reaction association rules is to encode them into the stoichiometric matrix of the GEM [37]. This encoding makes it possible to extend flux sampling approaches to gene sampling, facilitating the quantification of uncertainty. These sampling approaches are discussed further in the flux simulation section.

**Table 1:** Summary of approaches that address sources of uncertainty in genome annotation. Highlighted in bold are key approaches related to probabilistic or ensemble-based methods

Approach	Sources of uncertainty	References
Comparison of pipelines	Variability across databases	[19]
Combining databases	Variability across databases	[33, 34]
Template GEMs	Incomplete annotations in non-model organisms	[38–43]
**Probabilistic annotation**	**Annotation errors**	[44, 45]
**Probabilistic annotation + context Information**	**Annotation errors**	[46, 47]
Specific databases and high-throughput genomics	Annotation errors	[48–54, 56, 59–61]

A few reconstruction pipelines try to circumvent the problem of incorrect or missing functional annotation by using previously curated GEMs as annotation templates. Using several different reconstruction pipelines—RAVEN [38, 39], AuReMe/Pantograph [40, 41], or MetaDraft [42]—the user can map annotations from one organism directly to a curated model of a closely related organism by employing homology searches between the two. In this way, well-curated metabolic reaction annotations from an established GEM are propagated to new GEM reconstructions. Another reconstruction pipeline, CarveMe, uses a curated network of all possible reactions, based on the BiGG database [13], as the reference and “carves out” a subset of reactions to create organism-specific models [43]. While these methods may provide more complete reconstructions that require less gap-filling, they do not solve the fundamental issue of the uncertainty in the mapping of homologs or provide an estimate of the uncertainty associated with the presence of each reaction in the network.

Another approach is to directly incorporate uncertainty in functional annotation by assigning several likely annotations to each gene rather than picking the single most likely. In one likelihood-based approach, metabolic reactions are annotated probabilistically by taking into account the overall homology score, BLAST e-value, and keeping track of suboptimal annotations [44]. In this approach, metabolic reactions are assigned a probability of being present in a GEM based on both the strength and the uniqueness of the annotation. This approach has been developed into the ProbAnnoPy and ProbAnnoWeb pipelines that provide probabilistic annotations in the ModelSEED framework [45]. Beyond using only homology from BLAST to inform annotation probabilities, the CoReCo algorithm has additionally included homology scores based on global trace graphs, which have been proposed as an improved approach for identifying distant homologs [46]. The CoReCo algorithm also utilizes phylogenetic information to improve the probabilistic annotation of GEMs for multiple organisms simultaneously. Additional context information has also been incorporated into a probabilistic metabolic reaction annotation approach in the GLOBUS algorithm [47]. Context-based information includes gene correlations from transcriptomics, co-localization of genes on the chromosome and phylogenetic profiles, all of which are complementary to gene-sequence homology for inferring functional protein annotations. The probabilistic metabolic reaction annotations generated with these methods serve as a good starting point for subsequent reconstruction steps. For example, the likelihood-based approach mentioned here is used to implement a probabilistic gap-filling algorithm, further discussed in the gap-filling section [44].

Other concepts that have been used to generally improve gene functional annotation could be further incorporated into GEM annotation pipelines. For example, functional annotation of enzymes could be improved by the incorporation of enzyme active/catalytic site information from databases such as M-CSA [48]. Additionally, the annotation of specific classes of proteins, such as biosynthetic gene clusters [49, 50], transporters [51, 52], and amino acid biosynthetic pathways [53], can be improved by using approaches tailored to identify features that are specific to those protein classes. In particular, transport reactions are difficult to properly annotate and can add significant uncertainty to GEMs [14]. For example, the substrate specificity of automatically annotated transport reactions can often be improved with experimental data [54]. Furthermore, incorrect transport reactions can cause ATP generating cycles that lead to inaccuracies in GEM predictions [55]. Beyond traditional annotation approaches, machine learning has also been used to improve enzyme annotation by predicting EC numbers directly from gene sequences, potentially picking up on subtle features that would otherwise be missed by homology-matching-based approaches [56]. The localization of reactions to specific compartments is an added layer of annotation that is important for accurate GEM reconstruction, especially of eukaryotes [57, 58]. Also in this case, machine learning approaches can be used to predict the specific subcellular localization of proteins [59, 60]. New high-throughput genomics experimental methods can also be used to simultaneously assess the function of many genes in a large number of environments [54, 61]. Incorporating novel ideas from these methods into GEM reconstructions may reduce the overall uncertainty of functional annotation.

## Environment specification

To use a GEM for the prediction of expected phenotypes, or for the simulation of dynamic processes, one must define the chemical composition of the environment (Table 2). Establishing the list of environmentally available molecules is straightforward in simple laboratory experiments, in which defined media with known chemical composition are used. In this context, databases such as Media DB [62] or KOMODO [63] have cataloged a large number of defined media, greatly facilitating metabolic modeling. Many laboratory experiments, however, are performed in undefined media containing ingredients such as “yeast extract” that cannot be easily listed and quantified. In nature, microbes often exist in highly complex environments where the chemical inputs to the system are undefined, vary with time, and are altered by other microbes in the environment. Furthermore, it is not sufficient to know the list of compounds present in the cultivation medium, but one must also know at what rates the compounds can be consumed by the organism to properly set the bounds on the uptake reactions of the metabolic model. In principle, the composition of the environment can be determined through experimental techniques such as exo-metabolomics, where measurements of metabolites in the extracellular environment are used to infer cellular uptake and secretion rates [64–68]. This approach can provide valuable information for reducing the uncertainty in the environment specification. However, this data comes with its own uncertainty that should be carefully addressed [69]. All of these factors lead to a wide range of uncertainty arising in environment specification for metabolic network analysis [70].

**Table 2:** Summary of approaches that address sources of uncertainty in environment specification. Highlighted in bold are key approaches related to probabilistic or ensemble-based methods

Approach	Sources of uncertainty	References
Media databases	Inconsistent media definition	[62, 63]
Experimental determination	Undefined environment composition	[64–68]
Phenotype phase plane	Variable environment composition	[71, 72]
**Ensemble sampling**	**Variable environment composition**	[73–79]
**Probabilistic sampling**	**Variable environment composition**	[80]
Reverse ecology	Undefined environment composition	[81–85]

GEMs provide an opportunity to address the uncertainty associated with complex environments. GEM analysis algorithms, such as FBA, are computationally efficient and can thus be run across a large ensemble of environments to quantify the sensitivity of simulated fluxes to nutrient composition. Several studies have quantified this sensitivity by identifying aspects of GEM predictions that are either strongly affected by or robust to variation in the environmental composition [71–77]. Describing this sensitivity, or robustness, provides a clearer picture of how uncertainty in the environment specification may, or may not, propagate to specific GEM predictions. Early on, phenotype phase plane analysis was developed to show the impact on optimal growth rate of varying the fluxes of two limiting resources [71, 72]. Moving beyond pairs of resources, large ensembles of nutrients can be randomly sampled to assess the variability of all intracellular fluxes. For example, Almaas et al. showed, using a well-curated *Escherichia coli* GEM, that the overall distribution of metabolic fluxes is robust to the environmental composition; however, specific fluxes vary, with most discrete variations occurring in a connected “high-flux backbone” of reactions [73]. Subsequent work highlighted the evolutionary importance of an active core of reactions that carry flux in all environments [74]. Reed and Palsson further demonstrated that reactions with correlated fluxes across environments are indicative of transcriptional regulatory structure [75]. These studies point to the non-trivial nature of the sensitivity of GEM predictions to environment specification. Beyond the context of individual organisms, GEM analysis has been used to demonstrate that varying the environment can alter the nature of metabolic interactions between microbial organisms [78] and that certain environmental variables, such as the presence of oxygen, can have a significant impact on the interaction types that arise [79]. Variable environments can impact cellular metabolism from individual reaction fluxes up to the level of microbial interactions. Thus, in applications where the environment is uncertain, ensemble or probabilistic approaches are needed to fully capture potential phenotypes.

A more recent approach, inspired by the statistical physics concept of network percolation, utilizes random sampling of nutrient compositions to quantify which metabolites can be consistently produced by a given metabolic network across many environments [80]. This approach introduced a probabilistic framework for representing the input metabolites of a metabolic network, which could further facilitate random sampling of environmental ensembles in future methods. While the current implementation of this framework samples all environmental metabolites with equal probability, one could envisage future approaches which represent environmental uncertainties more accurately by using biased distributions that incorporate any available knowledge. This approach would fill the existing gap between assuming a single known environment and randomly sampling environments uniformly. Additionally, environment sampling could be used to vary the flux (in FBA) or concentrations (in dynamic FBA) of different environmental components, in addition to their presence and absence, to assess the impact of these quantities on metabolic network properties.

The specification of the environment for GEM analysis could be further improved using “reverse ecology” methods that aim to infer the native environment from the metabolic network structure either through constraint-based optimization [81–83] or by defining “seed” metabolites that are needed as inputs for a metabolic network and are therefore more likely to be found in that organism’s natural environment [84, 85]. Since these methods utilize the metabolic network structure to inform the environment specification, they should be applied carefully as uncertainty in the network may propagate into environment specification.

## Biomass formulation

The cell biomass used in GEMs is an inventory list of all compounds essential for growth of a given organism, weighted to represent the amount of each component present in 1 g of dry-weight biomass. The reaction that transforms all biomass components into a unit of biomass is used to represent growth in GEMs and is necessary to perform popular analyses such as FBA. Since several aspects of the biomass reaction and its use have been reviewed before [86], we will focus on the uncertainty associated with its formulation (Table 3).

**Table 3:** Summary of approaches that address sources of uncertainty in biomass formulation. Highlighted in bold are key approaches related to probabilistic or ensemble-based methods

Approach	Sources of uncertainty	References
Alternative biomass formulations	Variability in biomass within organisms	[96–101]
Environment-dependent biomass formulation	Variability in biomass within organisms	[102]
Cross-organism biomass comparison	Biomass differences across organisms	[87, 88]
Experimental determination	Undefined biomass composition	[103–105]
**Ensemble sampling**	**Undefined biomass composition**	[106]

The main source of uncertainty in the formulation of biomass composition is the lack of direct experimental measurements for most organisms. In the absence of specific data, the biomass composition from a model organism (e.g., *E. coli* for Gram-negative or *Bacillus subtilis* for Gram-positive bacteria) is often used as template, despite the significant uncharacterized variation in biomass composition likely to exist across different organisms. This trend has been verified by hierarchical clustering of biomass compositions from 71 curated GEMs: rather than taxonomic relations, the clusters were defined by the template biomass functions used in the model reconstruction [87]. Similarly, in a survey of plants, the biomass was only experimentally determined in 5 of 21 GEMs [88]. Furthermore, even within the same organism, the biomass composition can change in response to changes in growth rate, nutrient availability, temperature, and osmotic stress [89–95].

A number of studies have addressed the sensitivity of model predictions to changes in biomass formulation. Because these studies differ both in how the biomass function is changed and which model predictions are evaluated, they reach different conclusions. Initially, Pramanik and Keasling used correlations between growth rate and macromolecular abundances to estimate growth-rate-specific biomass compositions in *E. coli* [96, 97]. When the high growth-rate biomass composition was used to simulate fluxes in a low growth-rate environment, or vice versa, the total deviation from measured fluxes increased drastically compared to simulations with correct biomass specification [96]. Secondly, they showed that the predicted fluxes were sensitive to quantitative changes in the fatty acid composition of the biomass [97]. More recent analyses of the effect of changing the biomass composition in *Saccharomyces cerevisiae* have shown large influence on gene knock-out growth predictions [98], variable effect on substrate uptake rates [99], and an effect on the flux distribution dependent on the identity of the limiting nutrient [100]. In contrast, little effect was found on the predicted growth yield in *Pseudomonas putida* [101]. To address the dependence of the biomass formulation on the environment, within an individual organism, Schulz et al. propose two concepts for the incorporation of, or interpolation between, multiple biomass functions corresponding to different growth environments [102]. The first concept allows the GEM to choose an optimal linear combination of existing biomass functions while the second concept uses a hyperplane interpolation to predict the correct biomass function for the selected growth environment. The authors use hypothetical biomass functions to show that the choice of method has a clear impact on model predictions, but further evaluation calls for experimental follow-up. Swapping the biomass between different organisms can provide insight into the sensitivity of GEMS to strain specific biomass formulations, which is an important consideration given the widespread use of template biomass formulations. Leveraging three independent reconstructions of *Arabidopsis thaliana* with substantially different biomass reactions, it was found that the fluxes in central carbon metabolism were robust to replacement of the biomass reaction from one of the other models [88]. In contrast, swapping biomass reactions between five different bacterial species resulted in up to 30% change in predicted essential reactions [87].

Although the effect of uncertainty and error in the biomass coefficients depends on a large number of variables and how the effect is measured, it is clear that GEMs would benefit from increased precision in the estimation of biomass coefficients, which would ideally be organism and condition specific. The need for accurate estimates of the biomass composition has recently been addressed by experimental protocols [103–105] and the software BOFdat [106]. BOFdat provides a pipeline for computation of biomass coefficients and reports that the macromolecular composition is the most important factor in determining stoichiometric coefficients and should therefore be prioritized above ‘omics datasets. One elegant feature of BOFdat is a genetic algorithm which samples ensembles of biomass formulations to identify carbohydrate and small-molecule compositions such that model simulations optimally correspond with knock-out phenotype data. Looking forward, approaches such as BOFdat could be used to represent uncertainty in the biomass composition by sampling from an ensemble of possible biomass equations. Likewise, uncertainty in the stoichiometry of each biomass component could be incorporated by probabilistically sampling each coefficient from an appropriate distribution. Experimental data could be incorporated into this process to guide and constrain the distributions that are sampled through a Bayesian approach.

## Network gap-filling

Gap-filling is an important step in GEM reconstruction that transforms a draft network into one that can produce biomass in the specified environment (Table 4). The idea of gap-filling—that missing knowledge in metabolism may require algorithms to identify reactions absent in the representation of a specific pathway, but likely present in the organism—has been around since the early days of metabolic network modeling [107]. Gap-filling algorithms in general have been reviewed previously [108], but in brief, they utilize a universal database of possible reactions to augment an existing metabolic network with the goal of enabling feasible growth states, e.g., by connecting dead-end metabolites. Here we focus on the uncertainty associated with this process. Gap-filling is inherently uncertain because the reactions added are generally not supported by genomic evidence. Moreover, multiple solutions can often be found to satisfy the same gap-filling problem. Due to this uncertainty, basic gap-filling algorithms are known to be somewhat inaccurate [109], prompting recent benchmarking on randomly degraded metabolic networks to highlight the variability in gap-filling performance [110]. Furthermore, many GEMs contain significant inconsistencies even after the application of gap-filling approaches, and their identification is important for ensuring model fidelity [111].

**Table 4:** Summary of approaches that address sources of uncertainty in network gap-filling. While all gap-filling approaches address uncertainty arising from missing annotations, here we point out approaches that address uncertainty in the gap-filling solutions. Highlighted in bold are key approaches related to probabilistic or ensemble-based methods

Approach	Sources of uncertainty	References
Evaluating gap-filling accuracy	Degenerate solutions	[109, 110]
**Probabilistic gap-filling**	**Degenerate solutions**	[44, 45, 112, 113]
**Ensemble gap-filling**	**Degenerate solutions**	[43, 114–116]
De novo reaction prediction	Reaction database incompleteness	[121–124]

The uncertainty in gap-filling solutions has prompted the development of various probabilistic approaches to integrate data and prioritize solutions. An early innovation in probabilistic gap-filling algorithms was the development of a method to evaluate the addition of reactions to fill gaps based on a Bayesian network including sequence homology, operon, and pathway-based information [112]. A similar approach is to use probabilistic weights during the gap-filling process, such that more probable reactions incur a smaller penalty when added to the metabolic network. The CROP algorithm is an example of gap-filling based on growth phenotype data that implements weights based on various sources of evidence, including manually curated experimental evidence, pathways known to be associated with an organism, thermodynamics, and probabilistic estimates of enzyme function [113]. Another probabilistic approach has been developed to translate sequence homology into the likelihood that a metabolic reaction is present in a given metabolic network (discussed in the “Genome annotation” section); these likelihoods can then be used as probabilistic weights during the gap-filling procedure [44, 45].

Beyond probabilistic gap-filling methods, ensemble approaches have been developed to represent the uncertainty in gap-filling solutions as an ensemble of possible gap-filled GEMs. An early approach in this area prunes a universal metabolic network to identify locally minimal gap-filling solutions that align with experimental data [114]. In this approach, an ensemble of metabolic networks is generated by randomly assigning the order in which reactions are pruned from an original universal metabolic network. A similar pruning-based ensemble method, MIRAGE, additionally includes gene expression and phylogeny when weighting the order in which to remove reactions [115]. The idea of ensemble gap-filling was more fully developed by an approach that utilizes growth phenotype data in a randomized order to generate an ensemble of gap-filling solutions [116]. By randomly changing the sequence in which growth phenotype data was presented to the gap-filling algorithm, Biggs and Papin generated an ensemble of metabolic networks that equally agree with  the given data. This study further demonstrated that utilizing the ensemble gap-filling result can be more accurate than using the individual results, or a global simultaneously gap-filled result. An additional ensemble gap-filling approach is implemented in the CarveMe method. CarveMe generates ensembles of gap-filled models by assigning random weights to reactions without genomic evidence [43].

Finally, automated gap-filling methods are fundamentally limited by the underlying database(s) of metabolic reactions that they utilize [117, 118]. Thus, uncertainty in this database set can have a large impact on gap-filling performance. This is a major limitation when considering the complexity of the true metabolic universe and the fact that we likely do not know the proper annotations for all metabolic reactions. In light of this limitation, a number of methods have been developed to predict possible metabolic reactions based on general reaction rules. Many of these approaches have been reviewed previously in the context of predicting biosynthetic pathways for target compounds [25, 119, 120]. One of the earlier approaches, the BNICE framework, expands the metabolic universe by learning generic reaction rules from the KEGG reactome [121]. This framework was subsequently used to develop MINE and ATLAS, databases of theoretically possible compounds and enzymatic reactions, respectively [122–124]. BNICE also suggests three-level EC-numbers for hypothetical reactions, which can guide discovery of proteins associated with de novo reactions. The theoretical number of reactions in the expanded ATLAS is more than 10-fold higher than the number of reactions in KEGG, indicating that a large number of unexpected chemical transformations may be involved in metabolism. As we grapple with uncertainty in metabolic network reconstruction, de novo methods such as these can help us address unknown unknowns and provide exciting unanticipated insights. Moving forward, a combination of probabilistic and ensemble methods for data integration and de novo reaction prediction will enable the generation of gap-filled metabolic networks that represent uncertainty and can be better used to guide model refinement.

## Flux simulation

One of the most common and powerful uses of GEMs is the prediction of metabolic phenotypes at steady state through the computation of expected fluxes through each reaction. Because the rank of the stoichiometric matrix is almost always less than the number of reactions, the linear system of equations associated with steady state is, in general, underdetermined. Thus, there are an infinite number of solutions within the multidimensional solution space (a space where each dimension corresponds to the flux of a metabolic reaction) [125]. Any point within the solution space is a feasible solution representing a metabolic phenotype. While there often is an emphasis on identifying the *correct* solution in this solution space (i.e., an individual point closest to the outcome of experimental measurements), choices and uncertainty in some of the above aspects of the computation necessarily lead to uncertainty in the prediction of the fluxes themselves. In this section, we will review prior work addressing this uncertainty, with an emphasis on methods geared towards embracing and reporting it (Table 5).

**Table 5:** Summary of approaches that address sources of uncertainty in flux simulation. Highlighted in bold are key approaches related to probabilistic or ensemble-based methods

Approach	Sources of uncertainty	References
Alternative objective functions	Undefined cellular objective	[130–132, 134, 135]
Suboptimal solutions	Undefined cellular objective	[136–138]
Characterization of optimal solutions	Degenerate otimal solutions	[141–145]
Characterization of steady-state solution space	Degenerate solution space	[146–149]
**Random sampling**	**Degenerate solution space**	[151–160]
**Random sampling with probabilistic biases**	**Degenerate solution space**	[161, 162, 165]
Added constraints	Degenerate solution space	[55, 168–174, 177–179, 182–185]
Relaxed steady-state assumption	Steady-state assumption	[188, 189]

The flagship method for simulating metabolic fluxes in GEMs, FBA, uses linear programming to identify a point (or a subspace) in the solution space that optimizes a predefined cellular objective [23, 126–129]. Quite often, this objective is chosen to be the maximization of biomass production. A fundamental question that has surrounded the FBA approach since its early days is whether and under what conditions the assumption that biological systems operate close to a predictable optimum is valid, and if so, which objective function best represents the metabolic goals of a cell. Several studies have explored this uncertainty associated with the choice of the objective function. Schuetz et al. show that intracellular fluxes can be accurately predicted using FBA and an appropriate cellular objective [130]. However, none of the 11 selected objectives could provide the best predictability across different conditions when comparing predicted fluxes with ^13^C flux experiments in *E. coli*. It was early on demonstrated that FBA with maximization of growth rate could predict the phenotype of *E. coli* wild-type strains, supporting the assumption that unicellular organisms have evolved towards maximal growth [131]. Indeed, by minimizing the deviation from measured fluxes in yeast, maximization of growth rate was identified as the most likely objective in glucose-limited conditions [132]. Taking an inverse FBA approach, Zhao et al. predicted the objective function for *E. coli* strains evolved through 50,000 generations [133]. Although they identified an infinite number of objective functions that could describe the measured flux ratios, maximization of biomass alone was not one of these objectives [134]. A different study of these *E. coli* strains also provided nuance to our understanding of evolutionary pressures by confirming that *E. coli* evolves *towards* maximization of growth rate primarily by increasing substrate usage, but only if the ancestral strain is initially far from the optimum [135].

In a number of instances, the phenotypes of knock-out mutants are actually more accurately predicted when taking into account suboptimal solutions (near but not exactly on the FBA predicted optimum). For example, the increased accuracy of the MOMA and related methods stems from the assumption that a knock-out strain is still steered towards the wild-type optimum by the cellular regulatory network and may not necessarily approach the knock-out optimum [136]. The PSEUDO method can further improve the accuracy of knock-out flux predictions by assuming that the knock-out flux is closest to a degenerate space of suboptimal solutions near the wild-type optimum, representing regulatory variability around the wild-type solution [137]. The optimality of solutions has been further investigated in a study leveraging ^13^C-measurements of 9 different bacteria, which found  that metabolism operates close to a Pareto surface that balances the trade-off between maximization of growth and ability to adapt to changing conditions [138]. In summary, these results suggest that suboptimality may provide increased robustness to stochastic variation and perturbation, a property with known importance in biological systems [139, 140].

To avoid biased assumptions of the metabolic goal of a microorganism, one can characterize the complete solution space to describe all possible phenotypes satisfying the steady-state and flux constraints. It is important to note that, even at the optimum predicted by FBA, the solution is rarely unique. The predicted flux vector must therefore be analyzed with caution. Flux variability analysis (FVA) can be used to estimate the range of possible fluxes at the optimum [141], but since the range of each reaction is estimated independently, the method provides no information on the correlations between fluxes. More sophisticated methods include enumeration of alternative optima [142–145], or a full description of the solution space through flux coupling [146], extreme pathway analysis [147], elementary flux modes (EFMs) [148], and elementary flux vectors (EFVs) [149]. EFMs decompose the steady-state solution space into characteristic support minimal vectors, while EFVs have the added benefit of incorporating flux bounds to further constrain the space to a polyhedron. Although these methods provide an unbiased framework for identifying metabolic pathways, a representation of the entire solution space is generally intractable for genome-scale models because of the non-polynomial scaling with the number of reactions [150].

Random sampling provides a scalable approach to describe possible phenotypes in the solution space. Monte-Carlo-based algorithms [151–153] have proven useful for a large number of applications [154], from a general description of the distribution of metabolic fluxes [73, 155, 156] to transcriptional regulation of key enzymes [157] or comparison of bacterial strains [158]. However, verification of convergence is a key quality control of random sampling results currently lacking in analysis of GEMs [159]. The computational time required to reach convergence is a practical issue for large models, but recent work shows that the sampling results can be estimated at a reduced cost by using analytical methods and Bayesian inference [160]. Random sampling of the flux space can also be probabilistically biased to better represent uncertainty. A recent concept estimates the probability distribution of flux states that maximizes entropy with an average growth rate equal to the experimental value [161, 162]. As stated in the principle of maximum entropy, this probability distribution is the best representation of available knowledge [163, 164]. Another recently developed approach, Bayesian FBA, can be used to sample metabolic fluxes from a truncated multivariate normal distribution with prior distribution centered around zero [165]. In Bayesian FBA, prior knowledge such as measured growth and uptake rates, or ^13^C - flux data, can be elegantly incorporated in calculations of posterior flux distributions in a generic Bayesian framework that provides insight into the uncertainty associated with individual fluxes and flux couplings.

The uncertainty in model predictions can be reduced by introduction of additional constraints which reduce the size of the solution space [3, 125]. The most common constraints are those associated with limits on nutrient uptake (as defined by the environment composition), thermodynamic irreversibility, and the presence of specific reactions, such as the growth and non-growth associated maintenance [166, 167]. However, these constraints have their own associated uncertainties. Uncertainty in growth and non-growth associated maintenance derives both from the experimental growth data used to estimate these values [14], and variability in the maintenance cost of cellular processes in different environments and organisms [168]. The impact of this uncertainty on GEM predictions has only been briefly touched upon [169, 170]. Taking into account thermodynamic constraints on metabolic reaction fluxes is a powerful approach to improve model predictions, both by identifying subnetworks violating the second law of thermodynamics and to infer the direction of metabolic reactions from the calculated change in Gibbs free energy [55, 171–174]. However, the calculation of Gibbs free energy for the large number of reactions present in GEMs requires approximate approaches, such as the group contribution method [175, 176].

Another branch of methods uses either transcriptome [177] or proteome [178, 179] data to constrain reaction fluxes according to the abundance of proteins catalyzing the respective metabolic reactions. While transcriptomics data have the benefit of increased coverage of genes compared to proteomics (e.g., covers 60% of the enzymes in the yeast-GEM) [178], the transcript levels do not necessarily correlate with enzyme abundance [180, 181]. This may explain why Parsimonious enzyme usage FBA (pFBA), which minimizes the total sum of the absolute values of fluxes [182], in general outperformed seven different transcriptome-based methods in predicting intracellular fluxes for both *S. cerevisiae* and *E. coli* across three different conditions [177]. An additional advantage of pFBA is that it does not require additional parameters, unlike the aforementioned transcriptomics/proteomics approaches, which may require a large number of parameters to properly integrate the data. Similar to pFBA, several other methods use global constraints to improve model predictions. Of particular interest are Constrained Allocation Flux Balance Analysis (CAFBA) [183] which takes the growth-dependent ribosome allocation into account, the global constraint of dissipation of Gibbs free energy [184], and the extension of pFBA to include reaction likelihoods [185]. In any of these methods, particularly those that use additional data and parameters, it is important to remember that additional data used to further constrain the flux space comes with its own associated uncertainty, which must be taken into account when integrating it into GEMs.

The steady-state assumption forms the basis of constraint-based analysis by requiring mass-balance of all intracellular metabolites and defines the solution space discussed throughout this section. This assumption is justified because transient changes in metabolite concentrations occur rapidly compared to environmental and regulatory perturbations, leading to rapid convergence to a quasi-steady-state where metabolite concentrations are constant [186, 187]. However, when considering the uncertainty in stoichiometric coefficients, particularly in the biomass function, the steady-state assumption is effectively relaxed [165, 188, 189]. The RAMP approach demonstrates that relaxing the steady-state assumption can lead to more accurate predictions of intracellular fluxes [189]. The RAMP solution converges to the FBA solution when the uncertainty in stoichiometric coefficients approaches zero, demonstrating that this is a more general approach. While only uncertainty in the coefficients of the biomass reaction is explicitly tested in this work, RAMP’s general framework is not limited to this case and can include uncertainty in reaction bounds or uncertainty in coefficients associated with protein allocation or thermodynamics.

## Discussion

In this review, we highlighted methods that use probabilistic approaches and ensemble modeling to represent the uncertainty associated with constraint-based reconstruction and analysis of GEMs. Formalizing the representation of uncertainty in GEMs would improve confidence in modeling results. Although we concede that this is a difficult task, we hope that this review will serve as a roadmap for how this issue can be further addressed. We maintain that ensemble approaches (which are in essence discrete representations of probability distributions) provide a strong framework that naturally captures the uncertainty arising from the many possible outcomes in each step of the reconstruction and flux analysis process (Fig. 1). A practical step moving forward is the development of a unified metabolic network reconstruction and analysis framework that provides a probabilistic ensemble of results. Such a framework would require further development of methods for the representation and analysis of GEM ensembles, such as the MEDUSA package [190], and continued development and integration of approaches that represent uncertainty encountered in each stage of the GEM reconstruction and analysis process. In future development of ensemble models of GEMs, one should keep in mind that this approach is not a panacea [191]. It will be important to accurately account for uncertainty in each step to avoid potential pitfalls, such as an increase in false positive predictions given the sparse nature of the stoichiometric matrix. For example, when incorporating de novo predicted reactions into network gap-filling algorithms, the probabilistic weighting of these reactions would need to be carefully tuned. Additionally, it will be important to further explore correlations between the results of the different steps in the reconstruction and analysis process to fully understand uncertainty in this framework. For example, probabilistic genome annotation and ensemble gap-filling can work synergistically to identify candidate genes for orphan metabolic reactions. Conversely, uncertainty in metabolic network structure could be propagated through methods that use the network structure to infer the biomass formulation (such as BOFdat) or environment specification (such as reverse ecology). It is also important to focus on understanding the sensitivity of modeling results to uncertainty in specific parameters or steps in the pipeline. Generating an ensemble of results can provide insight into which results are robust to uncertainty in different parameters or model choices. Furthermore, clustering and classifying ensembles of results with machine learning algorithms can provide insight into which areas of genome-scale modeling are particularly sensitive and should be targeted for uncertainty reduction [192]. Ultimately, capturing all of the uncertainty in GEM reconstruction and analysis in a single pipeline will be a difficult task, and an emphasis should be placed on transparency and reproducibility such that all of the assumptions employed by a particular approach can be easily accounted for [193]. The standardization of model quality control provided by MEMOTE is an important contribution in this direction [194]. A similar community-effort towards standardized assessment and reporting of GEM uncertainties, as has been recently suggested by Carey et al., would be similarly highly beneficial [195].

Multiomics data integration is an increasingly important application of GEMS as biological studies are now collecting and analyzing multiple sources of high-throughput data. GEMs can facilitate the integration of this data in a knowledge-based format that provides mechanistic insight [20, 196]. Approaches and challenges in integrating ‘omics data into GEMs have been reviewed previously, with a particular focus on the difficulty of precise data integration due to GEMs’ lack of kinetic information [197]. It is important to consider how best to represent ‘omics data such that they can be integrated into GEMs. In line with the main message of our review, Ramon et al. suggest that a Bayesian perspective can aid the integration of ‘omics data by taking into account the uncertainty in the metabolic network and experimental observations [197]. In this context, ‘omics data can be used to constrain both the prior and posterior distributions from which ensembles of GEMs are sampled. Furthermore, GEMs can be used to simulate disparate types of ‘omics data, even though the explicit calculation of likelihoods may be intractable. Thus, the use of “simulation-based” Bayesian inference approaches is a promising route for informing GEM structure and parameters from data [198]. However, scaling Bayesian approaches up to deal with the large space of possible GEM reconstructions is an open, exciting and challenging research direction.

While this review has been entirely focused on uncertainty in GEM approaches, it is also important to remember that future efforts will need to creatively address major open questions on how to integrate metabolic models with other layers of biological complexity and their associated uncertainties. Several methods have been proposed to extend the basis of GEMs to include some other layers, such as metabolism and expression (ME) models that incorporate the processes of gene transcription and translation [199] or dynamic FBA that can simulate time courses of metabolic processes such as microbial growth curves [186, 200, 201], and can be extended to include multiple organisms and spatial structure [202–206]. Moving beyond the steady-state assumption, approaches based on kinetic models of metabolism can predict the concentrations of metabolites and fluxes through individual pathways. Although these models require a large number of kinetic parameters, beyond those required by GEMs, several methods exist for inferring these parameters and representing their uncertainty [207–209]. Finally, whole-cell modeling can be used to simultaneously model multiple processes in the cell and gain comprehensive insight into cellular physiology [210, 211]. However, considerable uncertainty in the many parameters required for kinetic and whole-cell modeling continues to limit their broad application [212, 213]. Thus, as new modeling approaches arise, it is likely that genome-scale metabolic modeling, which strikes a productive balance between scalability and scope with many successful applications [5–11], will continue to play a key role in the landscape of mechanistic modeling of biological systems. Further embracing uncertainty in this field is an exciting opportunity to continue to improve the application of this modeling framework.

## Supplementary Information

**Additional file 1.** Review history.
