# Supplementary material for: Addressing uncertainty in genome-scale metabolic model reconstruction and analysis
Source: Genome Biol. 2021 Feb 18;22:64. doi: 10.1186/s13059-021-02289-z (PMC7890832; doi:10.1186/s13059-021-02289-z)
Supplement: Supplementary file 1 — Additional file 1. Review history. [file 13059_2021_2289_MOESM1_ESM.docx]

**1^st^ round**

**Reviewer 1**

Is the topic of the article timely and of interest to a wide range of readers?

- Yes

Is the article well written and presented in a logical way?

- Yes

Do the authors provide a useful synthesis of the topic?

- No: Examples from the field of metabolic and biomedical engineering that use of GEMs to predict engineering strategies or novel drug targets while acounting for uncertainty are missing (or underrepreented)

Do the authors provide insightful discussion of the future directions for the field?

- Yes: The discussion empahasizes the use of ensemble modeling to account for uncertainties in metabolic models. What falling a bit short is more specific suggestions ow to incorporate uncertainties in the model reconstruction or structure in metabolic modeling approaches. For instance, the authors suggest earlier to use homology scores as a measure for (un)certainty of the presence of a reaction. How could such a measure be considered in metabolic modeling. Are reactions with low probability simply flagged or penalized (would result in a bias)?The same question arises frorophan reactions introduced by gap-fillling. I don't expect an elaborate solution to this problem in this review but in the current version of the manuscript ways to incorporate such uncertainty scores in simulations are weakly addressed. The authors refer to memote as an important contribution to develop standards for genome-scale models. I encourage the authors to call here for a similar community driven approach to derive standardized solution for the incorporation of uncertainties in GEM construction/ simulations. In the second part of the discussion, the authors mention modeling approaches beyond simple stoichiometric models of metabolic reactions, such as ME and kinetic models. If possible, the authors should streess how the lessns learned in stoichioemtric modeling apply for these models as well. Lastly, a discussion on how or if uncertainties in GEMS can be reduced should be included.

Do any tables, figures or boxes that are present enhance the manuscript?

- Yes

Would the manuscript benefit from any additional tables, figures or boxes?

- Yes

Comments

Bernstein et al. present an excellent review detailing the different sources of uncertainties in constructing genome-scale models and in simulated flux solutions. They further summarize current solutions to deal with these uncertainties. The manuscript is of high relevance for the metabolic modelling community. I only have a few comments

Section "Genome annotation"

A table summarizing the different scoring approaches would be helpful for the reader.

Section "Environment specification"

L. 182-L. 186 "Subsequent work highlighted the evolutionary importance of an active core of r 183 eactions that carry flux in all environments (Eivind Almaas, Oltvai, and Barabási 2005). Reed and Palsson (2004) further demonstrated that reactions with correlated fluxes across environments are indicative of transcriptional regulatory structure (Reed and Palsson 2004)."

Please add an explanation how this information relates to resolving uncertainty in the definition of environmental conditions.

ll. 186-190: The sensitivity of microbial community simulations to environmental compositions contrasts the statement of the previous paragraph in which the authors refer to work from Almaas et al. stating that uncertainties about the actual environmental conditions do not significantly impact metabolic fluxes in a pure culture (one organism). This distinct robustness or sensitivity should be more strongly highlighted. The sensitivity is relevant for the study of microbial interaction in natural environments as well as for the design of synthetic microbial communities and hence solutions to this issues or suggestions would be beneficial.

l. 208: Experimental determination - whenever possible - should always be the method of choice even though measurements will always be erroneous to some extent. Accordingly, this possibility should be presented first.

Discussion:

l. 489: The authors are encouraged to call for an equivalent community approach for implementing uncertainty measures in GEM reconstruction and simulations.

Further points are given above.

Minor comments:

Citations: Some reference include the initials of the first authors (e.g., l. 43: Z.A. King et al.; l. 184 Eivind Almaas, ..), the majority omits those initials. The style should be harmonized acc. To the journal's specifications

Line 35: The abbreviation COBRA needs to be defined.

L. 39: "These methods are now used to study biological systems for many different applications, including metabolic engineering, human metabolism and biomedicine, and microbial ecology".

'in' is missing before metabolic engineering.

L. 49: of in DOE must not be capitalized.

L. 100: (5) is missing on the right-hand side of Figure 1.

L. 221: Gram needs to be capitalized. Add bacteria or prokaryotes

L. 256: What is meant with standardized protocols? The authors might add Long and Antoniewicz (doi: 10.1021/ac502734e) if the methods presented therein can be classified as standardized.

L. 351-52: Schuetz et al reference is given two-times.

**Reviewer 2**

Is the topic of the article timely and of interest to a wide range of readers?

- Yes

Is the article well written and presented in a logical way?

- Yes

Do the authors provide a useful synthesis of the topic?

- Yes

Do the authors provide insightful discussion of the future directions for the field?

- Yes: It can further be strengthened based on comments

Do any tables, figures or boxes that are present enhance the manuscript?

- Yes

Would the manuscript benefit from any additional tables, figures or boxes?

- Yes

Comments

In this work Segrè and colleagues have reviewed the potential uncertainties in genome-scale metabolic model reconstruction and analysis and highlighted some useful approaches to resolve them. Overall, this work is timely as the number of genome-scale models continue to grow exponentially and raises a very important but often overlooked issue - uncertainties in reconstruction and analysis. This work is well written as well. I have few comments which I hope would further improve the paper quality.

1. The authors have listed 5 major areas where the uncertainties could exist. I feel that there are further areas which are potentially missed here and would like them to carefully revisit this. Some examples of missed areas at the model reconstruction level are gene-protein-reaction association rule reconstruction, reaction compartmentalization, identification and inclusion of metabolite transporters. Similarly some missed areas at the analysis level are the uncertainty in selection of relevant objective function (although biomass maximization works well in most cases, it is not universally applicable).

2. Related to previous comment, while selection of relevant biomass could be uncertain, there are many other model parameters are uncertain as well. Such examples include estimation of GAM and NGAM and the use of additional relevant constraints (enzyme capacity, enzyme usage, gibbs energy).

3. Multi-omic data integration and analysis is useful application of genome-scale models. There also exist lot of uncertainties and I feel that the authors could consider including a section where they survey uncertainties and propose solutions.

4. While the understanding of proposed solutions to address uncertainties with the use of probabilistic/ensemble methods is straight forward in all cases, it is difficult to interpret how it would benefit in biomass uncertainty. Does this mean authors proposing to use multiple biomass equations (with varied components) or repeat simulations in a range of biomass coefficient values and generate a ensemble of solutions in each condition? Either of this could work better than existing norm, so it's better to clarify this.

5. I think the current solitary figure in the paper is bit oversimplified. I wish the authors could consider drawing multiple figures/illustrations/tables (section-wise) to clearly explain where and what are the uncertainties and how they could be resolved in a much more detailed manner. Additionally, they should consider providing tables/supplementary tables listing all potential uncertainties in each step - this will serve as a easy reference for readers.

**Authors’ response**

Reviewer #1: Bernstein et al. present an excellent review detailing the different sources of uncertainties in constructing genome-scale models and in simulated flux solutions. They further summarize current solutions to deal with these uncertainties. The manuscript is of high relevance for the metabolic modelling community. I only have a few comments

We are grateful to the Reviewer for the positive feedback, and for the excellent constructive comments, which we address in detail below.

Section "Genome annotation"

A table summarizing the different scoring approaches would be helpful for the reader.

Based on this comment and comment #5 from Reviewer #2, we have added a table to each section of the review which we believe will aid the reader by further organizing and summarizing the different approaches we cover. For the genome annotation section this table includes the different annotation scoring approaches.

Section "Environment specification"

L. 182-L. 186 "Subsequent work highlighted the evolutionary importance of an active core of r 183 reactions that carry flux in all environments (Eivind Almaas, Oltvai, and Barabási 2005). Reed and Palsson (2004) further demonstrated that reactions with correlated fluxes across environments are indicative of transcriptional regulatory structure (Reed and Palsson 2004)."

Please add an explanation how this information relates to resolving uncertainty in the definition of environmental conditions.

We appreciate the reviewer pointing out this potentially confusing section. We have added two sentences at the beginning of this section (Lines 220-224) to clarify our view of how the work demonstrated in these studies, quantifying the sensitivity of GEM predictions to environmental variability, relates to understanding the impact of uncertainty in environment specification. We have also added a sentence after the two references mentioned by the reviewer, describing how these references fit into this context (Lines 235-236). Furthermore, we have added several references to this section, including a discussion of phenotype phase planes to provide a more complete context for these approaches.

ll. 186-190: The sensitivity of microbial community simulations to environmental compositions contrasts the statement of the previous paragraph in which the authors refer to work from Almaas et al. stating that uncertainties about the actual environmental conditions do not significantly impact metabolic fluxes in a pure culture (one organism). This distinct robustness or sensitivity should be more strongly highlighted. The sensitivity is relevant for the study of microbial interaction in natural environments as well as for the design of synthetic microbial communities and hence solutions to this issues or suggestions would be beneficial.

Again, we appreciate the reviewer pointing out this section and clearly describing potential contradictions/confusion in our current explanation. As mentioned in response to the previous comment, we have attempted to clarify this section, by more clearly articulating the difference between quantifying robustness and sensitivity to environmental composition variation in two new sentences at the beginning of this section, and a discussion of phenotype phase planes to provide additional context (Lines 220-228). The references that we include here point to the fact that some GEM predictions can be sensitive to environmental perturbations (e.g. microbial interactions) while others are not (e.g. overall distribution of metabolic fluxes in an individual organism). We also agree with the reviewer that the sensitivity we describe is relevant for a number of applications. It is for this reason that we suggest that efforts to quantify uncertainty are a key step in improving the use of GEMs in these applications. We have attempted to highlight this further with additional text at the end of this section (Lines 240-242).

l. 208: Experimental determination - whenever possible - should always be the method of choice even though measurements will always be erroneous to some extent. Accordingly, this possibility should be presented first.

We agree with the reviewer’s point. We have moved the discussion related to experimental determination of nutrient uptake and metabolite secretion, that was previously at the end of the environment specification section, to the first paragraph (Lines 208-216). We have also added several references to this section that we believe are relevant, and serve to strengthen this point. Moving this section also allowed us to further elaborate on the “reverse ecology” concept described in the last paragraph of the environment specification section (Lines 255-262).

Discussion:

l. 489: The authors are encouraged to call for an equivalent community approach for implementing uncertainty measures in GEM reconstruction and simulations.

Further points are given above.

We appreciate this excellent suggestion. We have now included such a statement at the end of this paragraph and included an additional reference that emphasizes this point (Lines 587-589).

Minor comments:

Citations: Some reference include the initials of the first authors (e.g., l. 43: Z.A. King et al.; l. 184 Eivind Almaas, ..), the majority omits those initials. The style should be harmonized acc. To the journal's specifications

Thanks for noticing. We have removed all initials and first name from the citations in the text. We are aware that the Genome Biology use numbering and not author-year citation style, but we prefer to keep the author-year for review and editing purposes until the final formatting prior to publication.

Line 35: The abbreviation COBRA needs to be defined.

We now define the abbreviation (Lines 35-36).

L. 39: "These methods are now used to study biological systems for many different applications, including metabolic engineering, human metabolism and biomedicine, and microbial ecology".

'in' is missing before metabolic engineering.

Corrected as suggested (Line 40).

L. 49: of in DOE must not be capitalized.

Corrected to the US Department of Energy (Line 50).

L. 100: (5) is missing on the right-hand side of Figure 1.

We are unfortunately not able to understand this comment as there is not supposed to be any “(5)” in Figure 1. Anyway, based on reviewer 2 comment 5 we have edited the figure so we assume this issue is solved as well. If not, please get back to us with a more detailed description.

L. 221: Gram needs to be capitalized. Add bacteria or prokaryotes

Corrected as suggested by the reviewer (Lines 278-279).

L. 256: What is meant with standardized protocols? The authors might add Long and Antoniewicz (doi: 10.1021/ac502734e) if the methods presented therein can be classified as standardized.

As the reviewer correctly points out, standardized only adds confusion to this otherwise precise sentence which we now have edited by removing that adjective. We have also added the reference suggested by the reviewer. (Lines 320-321)

L. 351-52: Schuetz et al reference is given two-times.

Removed second reference to Schuetz et al. (Line 429)

Reviewer #2: In this work Segrè and colleagues have reviewed the potential uncertainties in genome-scale metabolic model reconstruction and analysis and highlighted some useful approaches to resolve them. Overall, this work is timely as the number of genomescale models continue to grow exponentially and raises a very important but often overlooked issue - uncertainties in reconstruction and analysis. This work is well written as well. I have few comments which I hope would further improve the paper quality.

1. The authors have listed 5 major areas where the uncertainties could exist. I feel that there are further areas which are potentially missed here and would like them to carefully revisit this. Some examples of missed areas at the model reconstruction level are geneprotein- reaction association rule reconstruction, reaction compartmentalization, identification and inclusion of metabolite transporters. Similarly some missed areas at the analysis level are the uncertainty in selection of relevant objective function (although biomass maximization works well in most cases, it is not universally applicable).

We thank the Reviewer for this insightful comment and suggestion of several aspects of GEM reconstruction and analysis that should receive additional attention in our manuscript. We address each of these areas below:

Gene-protein-reaction association rules - This is indeed a very important component of GEM reconstruction. We believe that this step is strongly related to genome annotation and have accordingly added text and several references related to gene-protein-reaction associations into the genome annotation section (Lines 124-138).

Reaction compartmentalization - We agree that reaction compartmentalization is an additional potentially important source of uncertainty, particularly in GEM reconstructions for eukaryotic organisms or when considering the modeling of communities. We have now added a discussion related to reaction compartmentalization and several references to the genome annotation section (Lines 185-189).

Transporters - We had briefly included the annotation of transporters in our review section on genome annotation. In the last paragraph we reference transportDB, a specific annotation database that can aid in the annotation of transporters and reduce uncertainty. We have also now added additional references and several sentences further describing the uncertainty associated with transport annotation (Line 175, 177-182).

Objective function - We believe that we have thoroughly discussed the uncertainty in selection of a relevant objective function. We included this discussion in the flux simulation section where we cover the choice of biomass production maximization as well as non-biomass related objectives (Lines 420-442). We have also now included an additional sentence to make the message of this paragraph clearer (Line 427-428).

2. Related to previous comment, while selection of relevant biomass could be uncertain, there are many other model parameters are uncertain as well. Such examples include estimation of GAM and NGAM and the use of additional relevant constraints (enzyme capacity, enzyme usage, gibbs energy).

We agree with the Reviewer’s comment that these additional constraints are important for addressing uncertainty. We have now included a concise discussion of maintenance costs, including several new references (Lines 500-505). Furthermore, we also include a discussion on the uncertainty associated with Gibbs free energy calculations (Lines 505-512) and we modified the existing text related to the incorporation of enzyme capacity and usage to further emphasize the reviewers point that the associated parameters themselves are uncertain (Line 526-529 and 533-534).

3. Multi-omic data integration and analysis is useful application of genome-scale models. There also exist lot of uncertainties and I feel that the authors could consider including a section where they survey uncertainties and propose solutions.

We appreciate this comment from the reviewer, and agree that multi-omic data integration is a useful application of genome-scale metabolic models and the uncertainty associated with these approaches deserves further attention in our manuscript. To this end, we have included an additional paragraph in the discussion to address this topic (Lines 590-604).

4. While the understanding of proposed solutions to address uncertainties with the use of probabilistic/ensemble methods is straight forward in all cases, it is difficult to interpret how it would benefit in biomass uncertainty. Does this mean authors proposing to use multiple biomass equations (with varied components) or repeat simulations in a range of biomass coefficient values and generate a ensemble of solutions in each condition? Either of this could work better than existing norm, so it's better to clarify this.

We appreciate that the reviewer raises this question. We believe that uncertainty in the biomass composition could be represented by multiple biomass equations with varied components. Likewise, uncertainty in the stoichiometric coefficients of the biomass equation could be represented by sampling these values probabilistically. Both approaches could be used to develop ensemble models that better represent uncertainty. We have tried to emphasize this point throughout our review and have updated the text at the end of the biomass section to try to make this clearer in that context (Lines 327-332).

5. I think the current solitary figure in the paper is bit oversimplified. I wish the authors could consider drawing multiple figures/illustrations/tables (section-wise) to clearly explain where and what are the uncertainties and how they could be resolved in a much more detailed manner. Additionally, they should consider providing tables/supplementary tables listing all potential uncertainties in each step - this will serve as a easy reference for readers.

We appreciate this comment from the reviewer. Upon considering multiple alternative options to address this point, we realized that the idea of tables summarizing the different approaches that we reviewed and the potential uncertainties at each step, as suggested by the reviewer, would be very valuable to help the readers orient themselves, and more easily find references and text for each topic. After generating these tables, we felt that additional step-specific figures would not be essential. Therefore, other than the left backbone of Fig. 1, illustrating in a very bigpicture overview the process along which the different steps occur, we removed other portions of the figure. As pointed out by the reviewer, the right-hand side of figure 1, was oversimplified and is now more clearly provided in the section tables. We also believe that the message from figure 1, the branching process leading to uncertainty, is more clearly conveyed by removing the right-hand side.
